# Ambient Availability of Amino Acids, Proteins, and Iron Impacts Copper Resistance of *Aspergillus fumigatus*

**DOI:** 10.3389/fcimb.2022.847846

**Published:** 2022-04-22

**Authors:** Annie Yap, Heribert Talasz, Herbert Lindner, Reinhard Würzner, Hubertus Haas

**Affiliations:** ^1^ Institute of Molecular Biology, Biocenter, Medical University of Innsbruck, Innsbruck, Austria; ^2^ Protein Micro-Analysis Facility, Institute of Medical Biochemistry, Biocenter, Medical University of Innsbruck, Innsbruck, Austria; ^3^ Institute of Hygiene and Medical Microbiology, Department of Hygiene, Microbiology, and Public Health, Medical University of Innsbruck, Innsbruck, Austria

**Keywords:** fungi, molds, *Aspergillus fumigatus*, copper, toxicity, amino acids, histidine, iron

## Abstract

The transition metals iron and copper are required by virtually all organisms but are toxic in excess. Acquisition of both metals and resistance to copper excess have previously been shown to be important for virulence of the most common airborne human mold pathogen, *Aspergillus fumigatus*. Here we demonstrate that the ambient availability of amino acids and proteins increases the copper resistance of *A. fumigatus* wild type and particularly of the Δ*crpA* mutant that lacks export-mediated copper detoxification. The highest-protecting activity was found for L-histidine followed by L-asparagine, L-aspartate, L-serine, L-threonine, and L-tyrosine. Other amino acids and proteins also displayed significant but lower protection. The protecting activity of non-proteinogenic D-histidine, L-histidine-mediated growth inhibition in the absence of high-affinity copper uptake, determination of cellular metal contents, and expression analysis of copper-regulated genes suggested that histidine inhibits low-affinity but not high-affinity copper acquisition by extracellular copper complexation. An increase in the cellular copper content was found to be accompanied by an increase in the iron content, and, in agreement, iron starvation increased copper susceptibility, which underlines the importance of cellular metal balancing. Due to the role of iron and copper in nutritional immunity, these findings are likely to play an important role in the host niche.

## Introduction

The mold *Aspergillus fumigatus*’ arsenal of nutrient-acquiring mechanisms allows its survival in the environment and diverse host niches, which makes this opportunistic pathogen the major cause of invasive pulmonary aspergillosis in immunocompromised patients worldwide (Latgé and Chamilos, 2019). In particular, the redox-active metals iron (Fe) and copper (Cu) have been previously shown to be important for its survival and virulence (Gerwien et al., 2018; Raffa et al., 2019; Misslinger et al., 2021). On the one hand, the redox potential makes Cu an excellent cofactor for many enzymes such as cytochrome oxidase (CoxB), superoxide dismutase (SodA), or laccases such as ferroxidase (FetC), which is involved in reductive Fe assimilation (Oberegger et al., 2000; Blatzer et al., 2011; Anabosi et al., 2021). On the other hand, the very same redox potential of Cu can result in toxicity as Cu catalyzes the formation of reactive oxygen species (ROS) *via* Fenton-like chemistry or cause mismetallation such as displacement of Fe in Fe–sulfur cluster-containing enzymes leading to their inactivation (Gerwien et al., 2018; Raffa et al., 2019).

Owing to this toxicity, Cu has been used for centuries in different chemical combinations as an antimicrobial agent against plant pathogens, as “self-sanitizing” Cu-alloy surfaces to prevent nosocomial infections, or as ointments to treat superficial infections of animals and humans (Festa and Thiele, 2011; Besold et al., 2016). Remarkably, Cu toxicity is also employed by the mammalian innate immune system to fight invading pathogens because massive amounts of Cu are pumped into the phagolysosome to support killing of phagocytosed pathogens (Gerwien et al., 2018).

Maintenance of Cu homeostasis in *A. fumigatus*, which has to ensure sufficient Cu supply in combination with avoidance of Cu toxicity, is based on a sophisticated transcriptional regulation. During Cu limitation, the Cu-sensing transcription factor Mac1 activates high-affinity Cu uptake mediated by the Ctr family members CtrA2 and CtrC (Park et al., 2014; Cai et al., 2017; Kusuya et al., 2017; Wiemann et al., 2017). Consequently, inactivation of Mac1 causes a growth defect under Cu limitation. On the other hand, the Cu excess-sensing transcription factor AceA activates Cu detoxification mediated mainly by cellular Cu export *via* the P-type ATPase CrpA (Wiemann et al., 2017; Cai et al., 2018). Consequently, inactivation of either AceA or CrpA increases the susceptibility of *A. fumigatus* to Cu. Both Mac1 and AceA have been shown to be important for *A. fumigatus* pathogenicity (Cai et al., 2017; Wiemann et al., 2017; Cai et al., 2018).

Mac1 was reported to also play a role in Fe regulation in *A. fumigatus* (Park et al., 2018), which could not be confirmed by us (Yap et al., 2020). In the latter study, we noticed that the nitrogen source used in the growth medium influences Cu resistance, i.e., Cu resistance was higher with glutamine (Gln) compared to nitrate. The aim of this study was thus to analyze the impact of nitrogen sources and ambient availability of amino acids (AAs), proteins, and Fe on Cu resistance.

## Materials and Methods

### Fungal Strains and Growth Conditions

If not otherwise stated, the *A. fumigatus* strain used was A1160, termed wild type (wt) here, and derived mutant strains Δ*mac1* (lacking Mac1), ΔaceA (lacking Ace1), and Δ*crpA* (lacking CrpA), which have been described previously (Cai et al., 2017; Cai et al., 2018). Furthermore, *A. fumigatus* strains Afs35 (a Ku70 lacking a derivative of the clinical isolate D141) (Krappmann et al., 2006), the clinical isolate Af293 (Nierman et al., 2005), Afs77 (a Ku70 lacking a derivative of the clinical isolate ATCC46645), and the Afs77-derived Δ*cccA* (Gsaller et al., 2012) and Δ*sidA* (Schrettl et al., 2004) mutant strains were used. The strains were grown at 37°C either on solid complex media (CM) or on/in solid/liquid Aspergillus minimal media (AMM) according to Pontecorvo et al. (1953) with 0.03 mM FeSO_4_ as Fe source (unless otherwise noted), 1% glucose as carbon source, and the nitrogen source described in the respective experiment. The Cu (CuSO_4_) concentration used is described in the respective experiments. For limitation of Cu or Fe, addition of the respective metal was omitted. CM contained 1% glucose, 2 g/l peptone (Carl Roth GmbH + Co. KG, Karlsruhe, S.T.U, Germany), 1 g/l casamino acids (Sigma-Aldrich Chemical Co., St. Louis, MO, USA), 1 g/l yeast extract (Lab M Limited, Bury, Lancs, UK), and trace elements according to Pontecorvo et al. (1953) but without Cu and Fe. Amino acid (AA) supplements are described in the respective experiment; if not noted otherwise, AAs were used in the L-configuration and were not denominated “L.” The nitrogen sources used were 20 mM ammonium (ammonium tartrate dibasic, (NH_4_)_2_C_4_H_4_O_6_), 20 mM Gln, 20 mM nitrate (sodium nitrate, NaNO_3_), 20 mM nitrite (sodium nitrite, NaNO_2_), and 20 mM urea. For plate growth assays, 1 × 10^4^ conidia were point-inoculated; AMM plates were incubated for 48 h at 37°C, and CM plates were incubated for 30 h at 37°C. For culturing in liquid medium, 100 ml AMM in 0.5-l Erlenmeyer flasks inoculated with 10^6^/ml conidia was shaken at 200 rpm at 37°C for 24 h. Bovine serum albumin and bovine pancreatic RNase A were from Sigma-Aldrich Chemical Co., St. Louis, MO, USA.

### Quantification of Cellular Cu and Fe Contents

The mycelia from liquid cultures were harvested by filtration, washed with distilled water, and freeze-dried to determine the dry weight of the biomass. For determination of the total cellular Fe content, 50 mg of freeze-dried mycelia was decomposed in closed polytetrafluorethylene vessels containing 2 ml of HNO_3_ and 0.5 ml of hydrogen peroxide using a high-performance microwave digestion unit (MARS 6, CEM Microwave Technology, Buckingham, UK). Appropriate dilutions were made with distilled water, and the total contents in Cu and Fe were determined by graphite furnace atomic absorption spectrometry (Zeeman GF95Z M6 AAS, Thermo Fisher Scientific, Waltham, MA, USA) according to standard methods.

### Northern Analyses

Total RNA was isolated according to the TRI Reagent (Sigma-Aldrich) method using peqGOLD PhaseTrap reaction tubes (PEQLAB, Erlangen, Germany). Formaldehyde-containing agarose gels were used to separate 10 μg of total RNA before blotting onto Hybond-N+ membranes (Amersham Biosciences, Amersham, UK) and hybridization with digoxigenin (Roche Diagnostics GmbH, Mannheim, Germany)-labeled probes. The digoxigenin-labeled hybridization probes used in this study were generated by PCR using primers 5′-ATGCGAACGAACATTGTCCC and 5′-CCAGCGGAAATGAGAAGATTCA for *crpA* (AfuA_3G12740), 5′-ATGGATCATATGAGCCAC and 5′-CTACCCGCAGCATTTG for *ctrC* (AfuA_2G03730), 5′-AAGCCGAGAAAAAGGGGG and 5′-AACCCGATGAA GCCCAG for *mirB* (AfuA_3G03640), 5′-ATATGTTCCTCGTGCCGTTC and 5′-CCTCAGTGAACTCCATCTC for *tubA* (Afu1g10910), 5′-GGAGCAGCTCGATCGCCAT and 5′-AGTGTATGCCACCATCGTTG for *atm1* (AFUA_6G12870), and 5′-CCCGTCTTCCACCTGCTG and 5′-GCATCAACAGCGCTGACCTT for *atm1* (AFUA_4G04318).

## Results

### Cu Resistance of *A. fumigatus* Is Influenced by the Nitrogen Source Used

**Figure 1:**
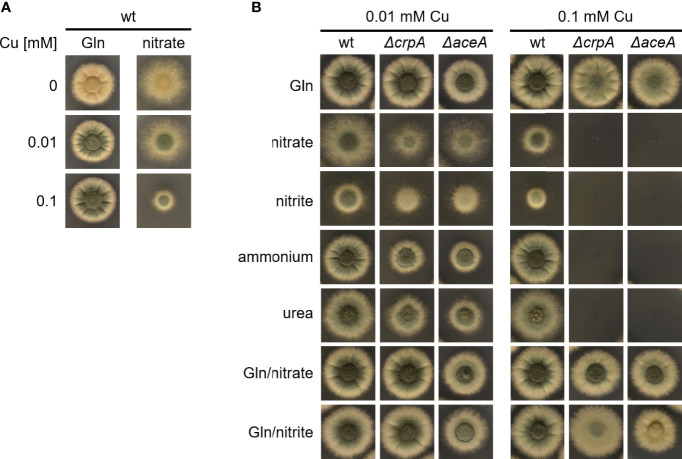
Gln protects *A*. *fumigatus* against Cu toxicity. **(A)** *A*. *fumigatus* wt conidia were point-inoculated on solid AMM containing 0, 0.01, or 0.1 mM Cu with either Gln or nitrate as nitrogen source. **(B)** To analyze the effect of the nitrogen source on Cu resistance, *A. fumigatus* wt, Δ*crpA*, and Δ*aceA* conidia were point-inoculated on solid AMM containing either 0.01 or 0.1 mM Cu in combination with different nitrogen sources (in case of combination of two nitrogen sources, the same concentration of each was used).

We observed that the Cu resistance of *A. fumigatus* wt is higher with 20 mM Gln compared to 20 mM nitrate as nitrogen source (**Figure 1A**): *A. fumigatus* A1160/*Δku80*, which is termed wild type (wt) here, displayed similar radial growth on solid media in the presence of 0.1 and 0.01 mM Cu as well as under Cu limitation with Gln as nitrogen source, while its radial growth decreased with increasing Cu concentration with nitrate as nitrogen source. Notably, Cu limitation is reflected by the yellow color of conidia, as biosynthesis of the green conidial pigment is dependent on a Cu-requiring laccase (Tsai et al., 1999). These results suggested that either glutamine protects against Cu toxicity and/or that nitrate enhances Cu toxicity. To test the latter hypotheses, we analyzed the impact of different nitrogen sources on Cu resistance of *A. fumigatus* (**Figure 1B**). To increase the sensitivity of the growth assay, we used not only *A. fumigatus* wt but also mutant strains that have increased susceptibility to Cu-mediated toxicity due to lacking transcriptional activation of Cu detoxification (*ΔaceA*, lacking the transcription factor AceA) or cellular Cu export (Δ*crpA*, lacking the Cu exporter CrpA), respectively (Raffa et al., 2019). The *ΔaceA* and Δ*crpA* mutant strains were able to grow in the presence of 0.1 mM Cu when using the nitrogen source Gln or Gln in combination with nitrate or nitrite. In contrast, the *ΔaceA* and Δ*crpA* mutant strains were unable to grow in the presence of 0.1 mM Cu with nitrate, nitrite, ammonium, or urea as nitrogen source. Moreover, *ΔaceA* and Δ*crpA* displayed significantly decreased growth compared to wt in the presence of 0.01 mM Cu in the absence of Gln. Taken together, these data indicate that the presence of Gln protects against Cu toxicity. In contrast to the other nitrogen sources used, the growth of wt decreased with nitrate and nitrite in the presence of 0.1 mM Cu compared to 0.01 mM Cu, which indicates that nitrate and nitrite might decrease Cu resistance.

### Supplementation With Different AAs Protects *A. fumigatus* Δ*crpA* Against Cu Toxicity to a Different Degree

**Figure 2:**
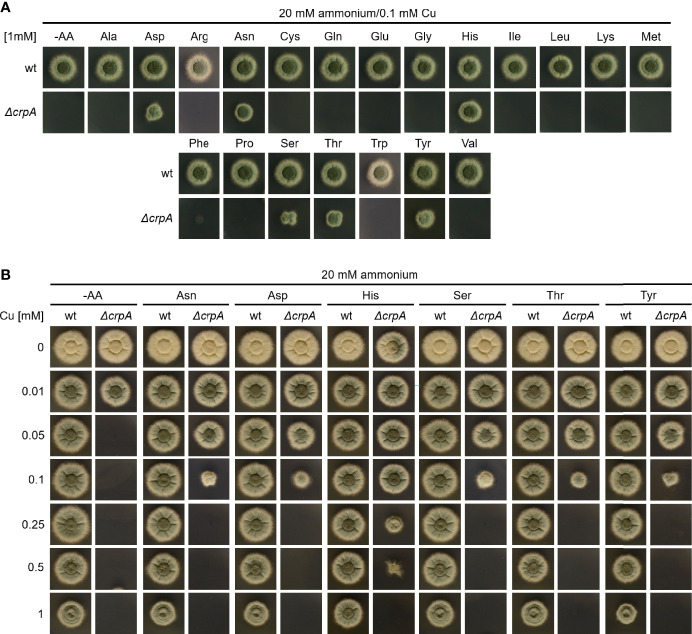
Supplementation with Asn, Asp, Ser, Thr, Tyr, and particularly His protects *A. fumigatus* Δ*crpA* against Cu toxicity to a higher degree compared to other AAs. *A. fumigatus* wt and Δ*crpA* conidia were point-inoculated on solid AMM containing 20 mM ammonium as nitrogen source. **(A)** The growth medium contained 0.1 mM Cu and was supplemented with 1 mM of the different AAs indicated (arginine, Arg; cysteine, Cys; glutamate, Glu; glycine, Gly; isoleucine, Ile; leucine, Leu; lysine, Lys; methionine, Met; phenylalanine, Phe; proline, Pro; tryptophane, Trp; valine, Val); -AA was without AA supplementation. **(B)** The growth medium contained 1 mM of the indicated AA and the different Cu concentrations indicated.

As Gln is an AA and as some AAs are well known for providing the ligands for metal binding in proteins (Cao et al., 2017), we analyzed the impact of supplementation with different proteinogenic AAs in a concentration of 1 mM on Cu resistance of *A. fumigatus* (**Figure 2**). Therefore, *A. fumigatus* wt and Δ*crpA* strains were grown on AMM plates containing 0.1 mM Cu and ammonium as nitrogen source, which impedes the growth of Δ*crpA* (**Figure 1**). Only supplementation with 1 mM asparagine (Asn), aspartate (Asp), histidine (His), serine (Ser), threonine (Thr), or tyrosine (Tyr) rescued the growth of the Δ*crpA* mutant strain, indicating that these six AAs have a higher Cu-detoxifying capacity compared to the other tested AAs such as Gln (**Figure 2A**). In order to identify possible differences in the Cu detoxification activity of these six AAs, we analyzed the growth of *A. fumigatus* wt and Δ*crpA* strains on media containing ammonium as nitrogen source, 1 mM of the respective AA, and different Cu concentrations up to 1 mM (**Figure 2B**). Supplementation with Asn, Asp, Ser, Thr, and Tyr rescued the growth of the Δ*crpA* mutant strain up to 0.1 mM Cu and with His up to 0.5 mM Cu demonstrating that His has the strongest protecting activity against Cu. Notably, the growth of wt was significantly decreased in the presence of 1 mM Cu compared to low Cu concentrations and exclusively His supplementation improved the radial growth of wt at this Cu concentration (**Figure 2B**).

**Figure 3:**
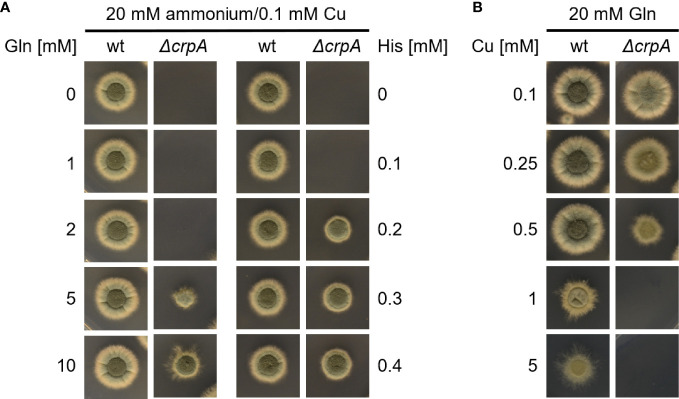
Supplementation with His protects *A. fumigatus* Δ*crpA* against Cu toxicity to a higher degree compared to Gln. *A. fumigatus* wt and Δ*crpA* conidia were point-inoculated on solid AMM. **(A)** The growth medium contained 20 mM ammonium as nitrogen source, 0.1 mM Cu, and different concentrations of either Gln (left) or His (right). The plate assay without Gln (0 mM Gln) is the same as the one without His (0 mM His). **(B)** The growth medium contained 20 mM Gln as nitrogen source and different concentrations of Cu.

In a next step, we compare the Cu-detoxifying activity of Gln and His. Determination of the AA concentration that is required to permit the growth of Δ*crpA* in AMM with ammonium as nitrogen source and 0.1 mM Cu yielded 5 mM Gln and 0.2 mM His (**Figure 3A**), which underlines the difference of a low- and high-protecting AA. Moreover, determination of the Cu detoxification capacity of 20 mM Gln, the standard nitrogen source used in our laboratory, yielded 0.5 mM Cu (**Figure 3B**).

Taken together, His showed the highest Cu-detoxifying capacity with an about twofold molar excess, i.e., supplementation with 1 mM His allowed the growth of Δ*crpA* in the presence of a maximum of 0.5 mM Cu (**Figure 2B**) and 0.2 mM His permitted the growth of Δ*crpA* in the presence of 0.1 mM Cu, respectively (**Figure 3A**). Asn, Asp, Ser, Thr, and Tyr detoxified Cu in an approximately 10-fold molar excess, i.e., 1 mM supplementation with either of these AAs allowed the growth of Δ*crpA* in the presence of a maximum of 0.1 mM Cu (**Figure 2B**). Gln detoxified Cu in an approximately 40–50-fold molar excess, i.e., 5 mM Gln was required to allow the growth of Δ*crpA* in the presence of 0.1 mM Cu (**Figure 3A**) and 20 mM Gln allowed the growth of Δ*crpA* in the presence of a maximum of 0.5 mM Cu (**Figure 3B**).

**Figure 4:**
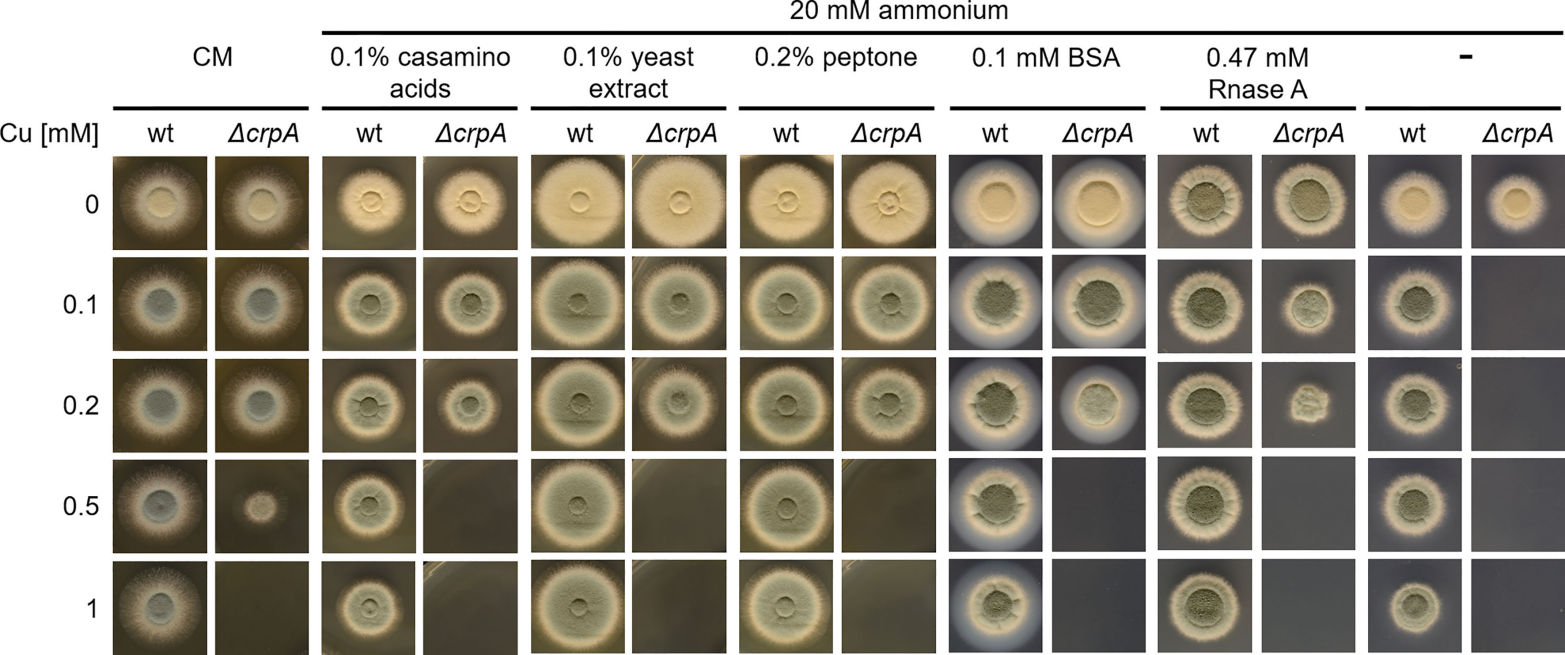
The complex medium ingredients casamino acids, yeast extract, and peptone as well as proteins protect *A. fumigatus* Δ*crpA* against Cu toxicity. *A. fumigatus* wt and Δ*crpA* conidia were point-inoculated on solid CM or AMM with ammonium as nitrogen source and supplemented with 0.1% casamino acids, 0.1% yeast extract, 0.2% peptone, or 0.1 mM BSA and different Cu amounts. The CM plates were incubated for 30 h at 37°C and the AMM plates for 48 h at 37°C.

Due to the Cu-detoxifying activity of AAs, we analyzed the Cu resistance of *A. fumigatus* wt and Δ*crpA* strains in complex medium (CM), which contains 0.2% peptone, 0.1% casamino acids, and 0.1% yeast extract. This medium allowed the growth of Δ*crpA* in the presence of a maximum of 0.5 mM Cu (**Figure 4**), which demonstrates its Cu-detoxifying activity. Each of the ingredients added to AMM with ammonium as nitrogen source improved the Cu resistance of Δ*crpA*, i.e., it allowed the growth of this mutant in the presence of up to 0.2 mM Cu (**Figure 4**). As these CM ingredients are not chemically defined components including AAs and peptides, we assayed in a next step the impact of a defined protein using bovine serum albumin (BSA). BSA supplementation to a final concentration of 0.1 mM also increased the Cu resistance of Δ*crpA* and allowed the growth of this mutant in the presence of up to 0.2 mM Cu (**Figure 4**). BSA consists of 583 AA residues, and consequently 0.1 mM BSA corresponds to 58.3 mM peptidic AAs. Therefore, at the level of AA molarity, BSA has a lower protecting activity compared to free AAs as 20 mM of the weakly protecting AA Gln allowed the growth of Δ*crpA* in the presence of up to 0.5 mM Cu (**Figure 3B**). Similar to BSA, supplementation with 0.47 mM RNase A, which consists of 124 AA residues and therefore corresponds to 0.1 mM BSA with respect to peptidic AA, allowed the growth of Δ*crpA* in the presence of up to 0.2 mM Cu, which underlines that the Cu resistance-promoting effect is a protein effect rather than being specific on the type of protein. Notably, the influence of proteins on Cu resistance might be influenced by proteolytic degradation of the proteins, but in the experiments conducted, proteolysis is expected to be low due to the presence of primary nitrogen (ammonium) and carbon (glucose) sources, which usually repress the expression of protease-encoding genes (Bergmann et al., 2009; Shemesh et al., 2017).

### His Supplementation Protects Against Cu Toxicity by Inhibiting Cu Uptake

**Figure 5:**
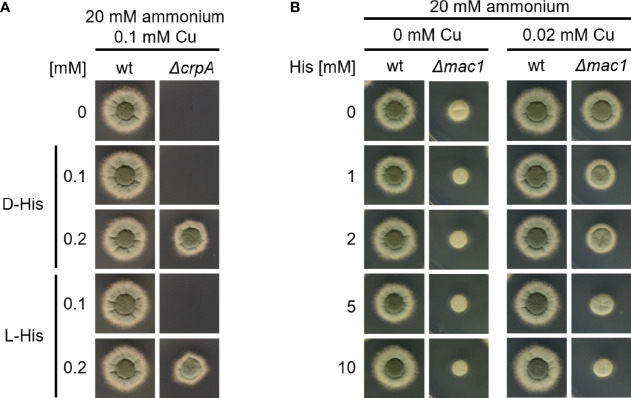
L-His and D-His show a similar protection of *A. fumigatus ΔcrpA* against Cu toxicity **(A)**, and His supplementation impedes Cu uptake by *A. fumigatus Δ*mac1. **(B)** *A. fumigatus* wt and mutant conidia were point-inoculated on AMM plates with 20 mM ammonium as nitrogen source. **(A)** The growth medium contained 0.1 mM Cu and was supplemented with different concentrations of either L-His (left) or D-His (right). **(B)** The growth media contained different concentrations of Cu and His, respectively.

To investigate the mechanism of how His supplementation protects *A. fumigatus* Δ*crpA* against Cu toxicity, we compared the protecting activity of His (L-His) and its non-proteinogenic stereoisomer D-His. As shown in **Figure 5A**, supplementation with L- and D-configurations of His resulted in similar Cu resistance, which indicates that the mode of protection conferred by His supplementation does not involve metabolization of His. Therefore, we hypothesized that His protects against Cu toxicity by extracellular chelation of Cu. In line, His supplementation was found to hamper the growth of the *A. fumigatus* Δ*mac1* mutant strain in a concentration-dependent manner under Cu-limiting conditions as well as in the presence of 0.02 mM Cu (**Figure 5B**). These data indicate that the presence of His negatively affects Cu uptake in the absence of high-affinity Cu uptake as Mac1 is essential for the activation of high-affinity Cu uptake (Raffa et al., 2019). In agreement, the growth of wt that is capable of high-affinity Cu uptake was not affected by His supplementation (**Figure 5B**).

To further investigate the impact of AA supplementation on metal homeostasis, we analyzed the cellular contents in Cu and Fe (**Table 1**). Therefore, *A. fumigatus* wt and Δ*crpA* strains were grown in liquid AMM containing 0.005 mM Cu and 20 mM ammonium as nitrogen source without AAs or supplemented with either 5 mM Gln or 1 mM His. The biomass production of wt was similar in all three growth media. In agreement with the Cu susceptibility of Δ*crpA* and the different Cu-detoxifying capacities of His and Gln, biomass of Δ*crpA* was only 22% of that of the wt in AMM without AAs, increased to 74% with Gln supplementation, and reached the wt level with His supplementation (**Table 1**). The cellular Cu content of wt was similar in AMM without AAs and with Gln but was approximately halved in the presence of His. In agreement with defective Cu export, the cellular Cu content of Δ*crpA* was increased about five-fold in the absence of AAs. Supplementation with His and Gln decreased the cellular Cu content to about that of the wt grown under the same condition (**Table 1**). Taken together, these data indicate that supplementation with AAs such as His and Gln protects *A. fumigatus* against Cu toxicity by impeding its cellular uptake, most likely *via* complexation of this metal. Remarkably, the about five-fold increase in cellular Cu of Δ*crpA* was accompanied by an approximately 10-fold increased Fe content (**Table 1**), which indicates a link between Cu and Fe homeostasis.

**Table 1:** AA supplementation impacts biomass production and cellular contents in Cu and Fe of *ΔcrpA*.

Strain	Supplement	Biomass ± STD[g]	Cu ± STD[µg/g]	Fe ± STD[µg/g]	Fe/Cu
**wt**	**-AA**	0.451 ± 0.008	53.5 ± 0.46	58.2 ± 0.41	1.1
	**+His**	0.512 ± 0.006	27.5 ± 0.62	72.1 ± 0.32	2.6
	**+Gln**	0.425 ± 0.004	55.4 ± 0.56	63.4 ± 0.46	1.1
***ΔcrpA***	**-AA**	0.101 ± 0.005	257.4 ± 0.78	604.8 ± 0.51	2.3
	**+His**	0.521 ± 0.008	30.6 ± 0.67	81.2 ± 0.37	2.7
	**+Gln**	0.313 ± 0.005	70.0 ± 0.79	69.3 ± 0.43	1.0

A. fumigatus wt and ΔcrpA strains were grown in liquid AMM containing 0.005 mM Cu and 20 mM ammonium as nitrogen source without AAs (-AA) or supplemented either with 5 mM Gln (+Gln) or 1 mM His (+His). After cultivation, dry biomass and cellular contents in Cu and Fe were determined after freeze-drying of the harvested mycelia. The shown values are the mean ± standard deviation (STD) of three biological replicates.

**Figure 6:**
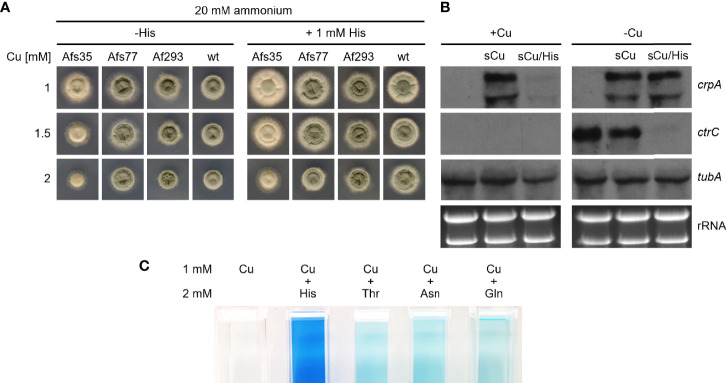
His supplementation protects *A. fumigatus* wt by inhibition of His uptake. **(A)** *A. fumigatus* conidia were point-inoculated on AMM plates with 20 mM ammonium as nitrogen source containing different Cu concentrations without (-His) and with 1 mM His supplementation. The plates were incubated for 48 h at 37°C. **(B)** *A. fumigatus* wt was grown for 16 h at 37°C in AMM liquid cultures with ammonium as nitrogen source without (-Cu; Cu limitation) and with (+Cu, Cu sufficiency) 0.005 mM Cu, respectively. Subsequently, the mycelia were harvested or incubated for another 45 min after addition of Cu to a final concentration of 0.2 mM without (sCu) or with (sCu/His) concomitant addition of His to a final concentration of 2 mM. Notably, Cu and His have been mixed before the addition to allow Cu–His complex formation. After harvesting the mycelia, total RNA was isolated and subject to Northern analysis of the indicated genes. Ethidium bromide-stained rRNA and α-tubulin encoding *tubA* served as controls for loading and quality of RNA. As observed previously (Wiemann et al., 2017), *crpA* has two transcripts, which are slightly larger and smaller as the 26S rRNA. **(C)** His, Thr, Asn, and Gln form colored complexes with Cu. The photos show cuvettes with 1 mM Cu or 1 mM Cu mixed with 2 mM of the indicated AA.

The experiments discussed above demonstrate the protective effects of AAs against Cu toxicity mainly for the highly Cu-susceptible Δ*crpA* mutant strain. As shown in **Figure 6A**, His supplementation also increases the radial growth of different *A. fumigatus* strains including the genetic background strain of the Δ*crpA* mutant, A1160, termed wt here. To further investigate the role of His in protection against Cu toxicity, we analyzed the expression of Cu-detoxifying ABC transporter-encoding *crpA* and high-affinity Cu uptake permease *ctrC* by Northern blot analysis (**Figure 6B**). During Cu sufficiency, neither *crpA* nor *ctrC* was expressed. A short-term confrontation (45 min) of such mycelia with 0.2 mM Cu caused an induction of *crpA* reflecting Cu detoxification. In contrast, a short-term confrontation with His-complexed Cu (the same amount of Cu was preincubated with a 10-fold excess of His before addition to the mycelia) did not induce *crpA*. The expression of *ctrC* was detected in neither of these mycelia, which confirms repression of high-affinity Cu uptake in agreement with Cu sufficiency. These data indicate that complexation of Cu by His blocks Cu uptake in the absence of high-affinity Cu uptake. During Cu limitation, the expression of *crpA* was repressed and *ctrC* was induced (**Figure 6B**). A short-term confrontation with Cu caused the induction of *crpA* and a decrease in the *ctrC* transcript level. In comparison, a short-term confrontation with His-complexed Cu induced *crpA* to the same degree as non-complexed Cu but repressed *ctrC* significantly more strongly. The similar induction of *crpA* by His-complexed and non-complexed Cu indicates that His complexation does not block Cu uptake by high-affinity systems. Moreover, the stronger repression of *ctrC* by His-complexed Cu compared to non-complexed Cu might indicate an even higher bioavailability for high-affinity uptake systems of His-complexed Cu compared to Cu alone. In agreement with complexation of Cu by AA, we observed that AA form blue-colored complexes with Cu; **Figure 6C** shows exemplary His, Thr, Asn, and Gln. His displayed the most intensive color formation with Cu, which is consistent with the strongest protecting activity observed. Notably, there was no significant difference in color formation between Thr, Asn, and Gln, which display different protecting activities at a lower level (see above).

### Fe Availability Impacts Cu Resistance and Vice Versa

**Figure 7:**
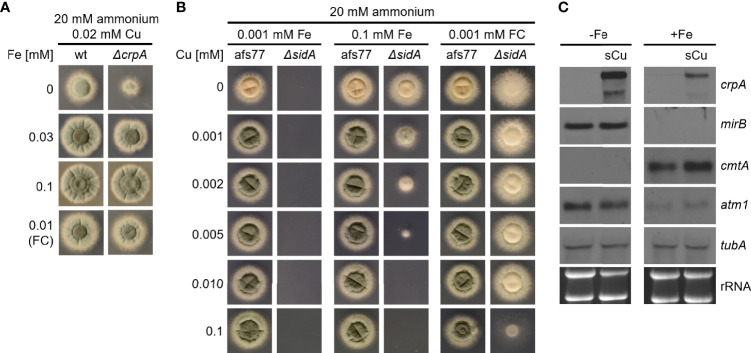
Fe availability impacts Cu resistance. **(A)** *A. fumigatus* conidia were point-inoculated on AMM plates with 20 mM ammonium as nitrogen source and 0.02 mM Cu combined with different concentrations of Fe or ferricrocin (FC)-chelated Fe. **(B)** *A. fumigatus* conidia were point-inoculated on AMM plates with 20 mM ammonium as nitrogen source with different concentrations of Fe or FC-chelated Fe combined with different concentrations of Cu. Afs77 is the genetic background of the Δ*sidA* mutant strain (Schrettl et al., 2004). **(C)** *A. fumigatus* wt was grown for 16 h at 37°C in AMM liquid cultures with ammonium as nitrogen source and 0.005 mM Cu without (-Fe; Fe limitation) or with (+Fe, Fe sufficiency) 0.03 mM Fe, respectively. Subsequently, the mycelia were harvested or incubated for another 45 min after addition of Cu to a final concentration of 0.2 mM (sCu). After harvesting the mycelia, total RNA was isolated and subject to Northern analysis of the indicated genes. Siderophore transporter encoding *mirB* was used as control for cellular Fe starvation (Schrettl et al., 2010). Ethidium bromide-stained rRNA and α-tubulin encoding *tubA* served as controls for loading and quality of RNA.

Based on the concomitant increase in cellular Cu and Fe contents in *A. fumigatus* Δ*crpA* (**Table 1**), we hypothesized that Fe is required to counteract Cu toxicity. Indeed, we found that the Cu resistance of *A. fumigatus* Δ*crpA* increases with increasing Fe availability (**Figure 7A**); i.e., in the presence of 0.02 mM Cu, the radial growth of Δ*crpA* was lowest during Fe limitation and increased with the degree of Fe supplementation. Furthermore, supplementation with ferricrocin-chelated Fe improved Cu resistance (**Figure 7A**). As Fe chelated by siderophores such as ferricrocin are taken up exclusively by siderophore-specific transporters (Aguiar et al., 2021; Misslinger et al., 2021), these data exclude the possibility that the positive impact of Fe supplementation on Cu resistance of Δ*crpA* is the sole consequence of competition of Fe with Cu for uptake by low-affinity metal transporters. In line, a Δ*sidA* mutant, which lacks siderophore biosynthesis and consequently displays decreased Fe acquisition (Schrettl et al., 2004; Misslinger et al., 2021), as shown by lack of growth under low Fe availability, shows decreased Cu resistance when grown with either 0.1 mM Fe or 0.001 mM ferricrocin (**Figure 7B**). The ferricrocin experiment again excludes the possibility that the effects seen are based on competition of Cu and Fe for uptake. Moreover, a short-term confrontation with Cu was found to induce a higher *crpA* expression during Fe starvation compared to Fe sufficiency (**Figure 7C**), which supports higher Cu toxicity under Fe limitation. To further investigate the role of Fe in Cu resistance, we analyzed the expression of *cmtA* (also termed *crd2*) and *atm1*. *cmtA* encodes a putative metallothionein previously implicated in the Cu resistance of *A. fumigatus* (Cai et al., 2018); *atm1* encodes a mitochondrial ABC transporter that links mitochondrial and cytosolic Fe–sulfur cluster biosynthesis and that has recently been shown to play a role in Cu toxicity in *Cryptococcus neoformans* and *S. cerevisiae* (Garcia-Santamarina et al., 2017). The expression of *cmtA* was found to be repressed under Fe starvation compared to Fe sufficiency and showed a slight upregulation in response to a short-term confrontation with Cu during Fe sufficiency (**Figure 7C**). In contrast, *atm1* displayed a downregulation during Fe sufficiency compared to Fe starvation without a response to a short-term confrontation with Cu (**Figure 7C**).

**Figure 8:**
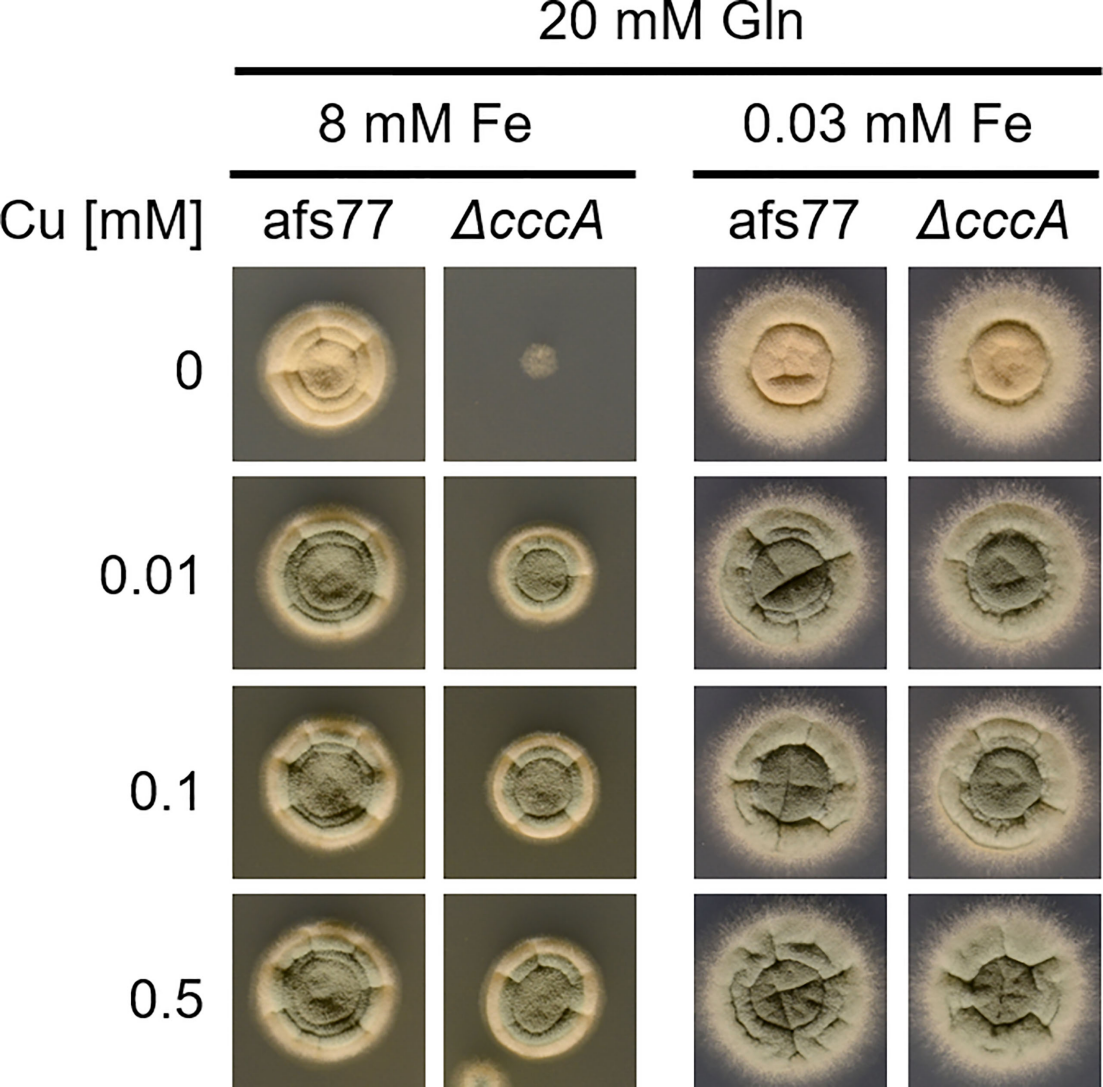
Cu availability impacts Fe resistance. *A. fumigatus* conidia were point-inoculated on AMM plates with 20 mM Gln as nitrogen source containing different concentrations of Cu and Fe. Gln was used here as nitrogen source as this high Fe amount precipitates with ammonium as nitrogen source. Afs77 is the genetic background of the Δ*cccA* mutant strain (Gsaller et al., 2012).

To investigate if there is also an effect of Cu availability on Fe resistance of *A. fumigatus*, we employed the Δ*cccA* mutant that shows increased Fe toxicity due to the lack of a vacuolar transporter mediating vacuolar Fe deposition (Gsaller et al., 2012). As shown in **Figure 8**, Fe resistance of the Δ*cccA* mutant increased with increasing Cu availability (8 mM Fe). Under low Fe availability (0.03 mM Fe), the Δ*cccA* mutant lacked a growth defect and displayed wt-like Cu susceptibility. Taken together, these data demonstrate the importance of metal homeostasis, i.e., balanced cellular metal contents.

## Discussion

In this study, we observed that the ambient availability of AAs and proteins increase the Cu resistance of the Cu-susceptible *A. fumigatus* Δ*crpA* mutant. Different AAs and proteins showed different protective activities in the order His > Asn ~ Asp ~ Ser ~ Thr ~ Tyr > Gln and other proteinogenic AAs > protein such as BSA and RNase A (**Figures 1** **–** **4**). Moreover, His supplementation also increased the Cu resistance of different *A. fumigatus* wild-type strains (**Figure 6A**). To adapt to different metal availabilities, fungal species employ both high-affinity and low-affinity transporters. High-affinity transporters display metal specificity and are induced under shortage of the respective metal, while low-affinity transporters usually show a broader metal specificity and ensure supply under conditions of high metal availability. The high-affinity Cu transporters of *A. fumigatus* are CtrA2 and CtrC (Cai et al., 2017; Kusuya et al., 2017; Wiemann et al., 2017). Low-affinity Cu transport has not been characterized in *A. fumigatus* yet, but this mold possesses a homolog of *S. cerevisiae* Fet4, a low-affinity transporter for Cu, Fe, and zinc (Hassett et al., 2000). Several lines of evidence indicated that His and most likely other AAs, although to a lower degree, increase Cu resistance by extracellular Cu complexation, which impedes uptake by low-affinity but not high-affinity systems: (i) non-proteinogenic D-His and His displayed similar protection of Δ*crpA* against Cu toxicity, indicating that the mode of action does not involve metabolization of His (**Figure 5A**); (ii) His supplementation caused dose-dependent growth inhibition of the *A. fumigatus* Δ*mac1* mutant that lacks transcriptional activation of high-affinity Cu uptake but not of the wild-type strain that is capable of high-affinity Cu uptake (**Figure 5B**); (iii) His supplementation decreased the cellular Cu content of Δ*crpA* (**Table 1**); (iv) Cu-sufficient mycelia, which displayed downregulation of high-affinity Cu uptake, responded to short-term exposure to Cu, but not to His-complexed Cu, with transcriptional downregulation of *crpA* (**Figure 6B**); (v) Cu-starved mycelia, which displayed upregulation of high-affinity Cu uptake, responded to short-term exposure to Cu and His-complexed Cu with transcriptional upregulation of *crpA* (**Figure 6B**); (vi) His supplementation is highly efficient in protecting Δ*crpA* against Cu toxicity most likely because high-affinity Cu uptake is downregulated in this mutant due to the high intracellular Cu content that represses Mac1 (Cai et al., 2018); and (vii) Cu was found to form blue-colored complexes with AAs, whereby the most intense color formation was found with His, which also displayed the highest-protecting activity (**Figure 6C**). In agreement with the latter, AAs are known to be able to form different chelates with Cu(II), whereby the metal-to-ligand molar ratio is 1:2 in the most common complex in aqueous solution (Deschamps et al., 2005). Among all AAs, His was able to complex Cu with the highest affinity. Consistently, His supplementation protected the Δ*crpA* mutant against Cu in an about two-fold molar excess (**Figures 2B**, **3A**). Remarkably, in response to short-term Cu exposure of Cu-starved mycelia, His complexation increased the transcriptional downregulation of the high-affinity Cu transporter *ctrC* compared to uncomplexed Cu, which indicates that His complexation improves the efficacy of Cu uptake by high-affinity transporters in contrast to low-affinity uptake systems (**Figure 6B**). Possibly, His complexation increases the bioavailability of Cu by increasing its solubility. In the Δ*crpA*, mutant strain, an about 5-fold increase in the cellular Cu content was found to be accompanied by an about 10-fold increase in the cellular Fe content (**Table 1**). This indicated an important role of cellular metal balancing. In agreement, several lines of evidence supported a role of Fe in protection against Cu toxicity: (i) increased Fe availability improved the Cu resistance of Δ*crpA* (**Figure 7A**), (ii) impaired Fe acquisition due to lack of siderophore biosynthesis decreased Cu resistance (**Figure 7B**), and (iii) short-term confrontation with Cu induced a higher *crpA* expression during Fe starvation compared to Fe sufficiency (**Figure 7C**). Vice versa, increased Cu availability was found to counteract Fe toxicity (**Figure 8**). These links between cellular Cu and Fe management might be explained by the fact that excess of a single metal might lead to mismetallation of proteins and/or that Fe and Cu are important for the detoxification of reactive oxygen species caused by excess of the respective other metal *via* Fenton/Fenton-like reaction, e.g., heme-Fe-containing catalases and peroxidases as well as Cu/Zn superoxide dismutase (Gerwien et al., 2018; Raffa et al., 2019; Brantl et al., 2021; Misslinger et al., 2021). Apparently, Fe does not decrease Cu toxicity *via* CrpA because Fe increased Cu resistance in both the absence (Δ*crpA*) and the presence of CrpA (Δ*sidA*) (**Figure 7B**). Recently, Fe–sulfur clusters have been shown to be targets for Cu toxicity in *C. neoformans* and *S. cerevisiae* and that the mitochondrial ABC transporter Atm1, which links mitochondrial and cytosolic Fe–sulfur biosynthesis, is transcriptionally upregulated in response to short-term exposure to Cu in *C. neoformans* but not *S. cerevisiae* (Garcia-Santamarina et al., 2017). We found that short-term exposure to Cu does not impact the expression of Atm1 at the transcript level in *A. fumigatus* (**Figure 7C**). Previously, genetic inactivation of the putative *A. fumigatus* metallothionein CmtA (also termed Crd2) was found to be dispensable for resistance to Cu as well as macrophage challenge (Wiemann et al., 2017; Cai et al., 2018). However, overexpression of CmtA in the absence of CrpA provided partial protection against Cu toxicity (Cai et al., 2018), indicating that CmtA plays a minor role in Cu resistance. Northern blot analysis demonstrated the repression of *cmtA* under Fe starvation compared to Fe sufficiency and a slight upregulation in response to short-term confrontation with Cu during Fe sufficiency (**Figure 7C**). These data might provide a hint for the role of Fe in Cu resistance. Nevertheless, it remains to be shown if CmtA is indeed a metallothionein. In line with the function as metallothionein, CmtA is a small protein rich in cysteine residues. However, previous studies indicated that both Cu availability and AceA do not impact *cmtA* expression; furthermore, the transcriptional Fe regulation is atypical for a metallothionein. Moreover, CmtA has been shown to physically interact with the monothiol glutaredoxin GrxD, which functions as a chaperon for distribution of Fe–sulfur clusters in the cytosol. Consequently, CmtA might have a role in Fe–sulfur cluster homeostasis.

The interaction of different metals in *A. fumigatus* has been reported previously. Due to the Cu dependence of reductive Fe assimilation (Askwith et al., 1994; Schrettl et al., 2004), impairment of Cu-independent siderophore-mediated Fe acquisition was found to increase the susceptibility of *A. fumigatus* to Cu starvation (Blatzer et al., 2011), and in line Cu starvation increases siderophore-mediated Fe acquisition (Yap et al., 2020). Moreover, Fe and zinc were found to be tightly linked: Fe starvation downregulates high-affinity zinc uptake and upregulates detoxification of zinc *via* vacuolar deposition in order to counteract zinc accumulation which displays higher toxicity during Fe starvation (Yasmin et al., 2009; Kurucz et al., 2018). In line, inactivation of the Fe regulator HapX was shown to impact zinc homeostasis (Schrettl et al., 2010) and Fe availability was reported to impact ZafA-mediated zinc regulation (Vicentefranqueira et al., 2019).

A crucial role of His in Cu handling has been previously noticed. For example, His auxotrophy combined with limited His supplementation was shown to decrease resistance to both starvation and excess of Cu in *A. fumigatus* (Dietl et al., 2016). Therefore, the avirulence caused by His auxotrophy (Dietl et al., 2016) might be a consequence not only of histidine shortage per se but also of metal mismanagement. Moreover, engineering of a *Saccharomyces cerevisiae* strain to display His oligopeptides at the surface increased Cu adsorption combined with increased Cu resistance (Kuroda et al., 2001). Moreover, it has been shown that His might decrease Cu toxicity also intracellularly under certain conditions in *S. cerevisiae* (Pearce and Sherman, 1999; Watanabe et al., 2014). AAs including Gln, Asn, Asp, Ser, and His were also found to play an important role in resistance to heavy metals including Cu in plants (Sharma and Dietz, 2006). Moreover, the human blood contains His-complexed Cu and the exchange of Cu(II) between His and albumin, which is able to bind Cu with high affinity and modulates cellular Cu availability (Deschamps et al., 2005; Sharma and Dietz, 2006).

In a process termed “nutritional immunity,” the mammalian innate immune system exploits the essentiality and toxicity of nutrient metals by producing factors that limit the availability of metals such as Cu and Fe to starve pathogens or intoxicate the pathogen with metal excess (Monteith and Skaar, 2021). Therefore, the impact of the ambient availability of amino acids and proteins on Cu resistance of *A. fumigatus* as well as the links between Cu and Fe homeostasis most likely play a role in the host niche. In particular, the combination of Cu excess with restriction of Fe, which aggravate Cu toxicity as shown here, appears to be a highly sophisticated defense strategy. Indeed, this combinatorial strategy is employed in the phagolysosome to attack pathogens: the antimicrobial activity of Cu is employed *via* import by the ABC transporter ATP7A (Gerwien et al., 2018), and Fe is exported by the transporter Nramp1 to deplete the phagolysosome of Fe needed by pathogens for growth (Forbes and Gros, 2001) and at the same time to aggravate Cu toxicity, as indicated by the data provided.

## Data Availability Statement

The original contributions presented in the study are included in the article/supplementary material. Further inquiries can be directed to the corresponding author.

## Author Contributions

HH conceived and supervised the study. HH and RW secured the funding of the study. HH, AY, HT HL, and RW designed the experiments. AY and HT conducted the experiments. HH and AY analyzed the data. HH and AY wrote the manuscript draft. All authors contributed to the article and approved the submitted version.

## Funding

This work was supported by the Austrian Science Fund (FWF) doctoral program “host response in opportunistic infections (HOROS, W1253 to AY, RW, and HH). We are grateful to Ling Lu (Jiangsu Key Laboratory for Microbes and Functional Genomics, College of Life Sciences, Nanjing Normal University, Nanjing, China) for providing some of the fungal strains used in this work. The funders had no role in study design, interpretation, decision to publish, in the writing of the manuscript, and in the decision to submit the manuscript for publication.

## Conflict of Interest

The authors declare that the research was conducted in the absence of any commercial or financial relationships that could be construed as a potential conflict of interest.

## Publisher’s Note

All claims expressed in this article are solely those of the authors and do not necessarily represent those of their affiliated organizations, or those of the publisher, the editors and the reviewers. Any product that may be evaluated in this article, or claim that may be made by its manufacturer, is not guaranteed or endorsed by the publisher.
